# A Neural Network Based Superstructure Optimization Approach to Reverse Osmosis Desalination Plants

**DOI:** 10.3390/membranes12020199

**Published:** 2022-02-09

**Authors:** Marcello Di Martino, Styliani Avraamidou, Efstratios N. Pistikopoulos

**Affiliations:** 1 Artie McFerrin Department of Chemical Engineering, Texas A&M University, College Station, TX 77843, USA; dimartino@tamu.edu; 2 Texas A&M Energy Institute, Texas A&M University, College Station, TX 77843, USA; 3 Department of Chemical and Biological Engineering, University of Wisconsin-Madison, Madison, WI 53706, USA; avraamidou@wisc.edu

**Keywords:** neural network modeling, surrogate modeling, reverse osmosis, mixed-integer linear programming

## Abstract

An ever-growing population together with globally depleting water resources pose immense stresses for water supply systems. Desalination technologies can reduce these stresses by generating fresh water from saline water sources. Reverse osmosis (RO), as the industry leading desalination technology, typically involves a complex network of membrane modules that separate unwanted particles from water. The optimal design and operation of these complex RO systems can be computationally expensive. In this work, we present a modeling and optimization strategy for addressing the optimal operation of an industrial-scale RO plant. We employ a feed-forward artificial neural network (ANN) surrogate modeling representation with rectified linear units as activation functions to capture the membrane behavior accurately. Several ANN set-ups and surrogate models are presented and evaluated, based on collected data from the H${}_{2}$Oaks RO desalination plant in South-Central Texas. The developed ANN is then transformed into a mixed-integer linear programming formulation for the purpose of minimizing energy consumption while maximizing water utilization. Trade-offs between the two competing objectives are visualized in a Pareto front, where indirect savings can be uncovered by comparing energy consumption for an array of water recoveries and feed flows.

## 1. Introduction

Due to a worldwide growing population the demand for water is ever-increasing, leading to global water scarcity that is not only driven by water quantity, but also by water quality issues [1]. The water supply systems are further stressed by climate change together with increased intensification of agriculture, industrialization, and water withdrawal [2,3,4,5], challenging the “clean accessible water for all” UN Sustainable Development Goal for 2030 [6].

Such stresses imply that conventional water sources are no longer sufficient to meet human water demands, especially in water-scarce regions [7]. Desalination is one of the technologies which can help overcome this challenge, since fresh or potable water can be obtained from available saline water sources [8,9]. Generally, desalination technologies can be classified based on either membrane processes or thermal separation [10]. The latter are highly energy-intensive and characterized by high capital and operational costs, due to their dependency on thermal energy, mainly produced from fossil fuels. On the other hand, membrane-based desalination processes are regarded as the most promising and practical desalination technologies, due to their high process energy efficiency [11]. Among membrane processes, reverse osmosis (RO) desalination is the industry leader constituting 69% of the worldwide installed desalination capacity, equalling $65.5\;M\frac{m^{3}}{\mathrm{day}}$ of produced fresh water [12]. Nevertheless, RO still remains an energy intensive process requiring process optimization, to minimize energy consumption [13,14].

The operation and design of brackish water reverse osmosis (BWRO) desalination plants has already been studied in the literature [11]. Ruiz-Garcia et al. for example evaluated operational windows of two BWRO systems based on hydrochemical fluctuations in well groundwater and derived optimal operational points in terms of minimum specific energy consumption and maximum water recovery [15]. Li studied the optimal operation of a BWRO desalination plant based on a constrained nonlinear optimization model resulting in a 10% energy consumption reduction while the same permeate flows are being maintained [16]. Further, Fellaou et al. analyzed the exergy of a full-scale BWRO desalination plant to analyze the performance of the main plant components to ultimately determine inefficiencies within the plant [17]. Additionally, Patel et al. used a rigorous system-scale RO model to determine the specific energy consumption and energy efficiency of the RO process over a wide range of brackish water conditions [18]. Kotb et al. optimized several multi-stage RO system arrangements identifying the cost minimizing operational parameters [19]. On the other hand, Sassi and Mujtaba combined the solution-diffusion model with film theory to derive a nonlinear optimization framework which minimizes the specific energy consumption of the RO system at fixed permeate output and quality based on operational parameters [20].

Generally, the optimization of RO systems requires modeling the mass transfer of the employed membrane modules [21], which may result in a complex mathematical model if first order principles are employed [22,23,24]; often a computationally expensive task [25]. Data-driven surrogate models can be utilized to capture the complex mass transfer behavior of membrane systems [26], utilizing machine learning (ML) methods [27]. ML can be classified into unsupervised and supervised learning, the difference between the two being that the former is used to analyze data with no apparent input-output connection, whereas the latter is used to obtain functions mapping an explicit input-output structure within the data [28]. Within supervised learning, the utilization of feed-forward artificial neural networks (ANNs) has been very popular [29], since ANNs have excellent approximation capabilities [30]. Given a sufficient number of neurons in only one hidden layer, ANNs can virtually approximate any function of interest to any degree of accuracy [31].

ANNs have already been applied to RO membranes and processes to analyze their respective performance [32,33,34,35]. Libotean et al. for example utilized an ANN surrogate model approach to estimate RO plant performance with applications in operational diagnostics [36]. Choi et al. employed surrogate models to analyze the long-term performance of full-scale RO desalination plants [37]. Sivanantham et al. modeled and optimized the rejection of chlorophenol in spiral wound RO modules using ANNs [38]. However, the data to train the ANN is either derived by model simulation [39,40], experimental single-stage RO membrane data points [41] or water sampling [42].

Furthermore, ANNs can be used for the operation optimization of membrane-based desalination systems, enabling effective decision making and better design [43,44]. Madaeni et al. modeled three RO systems with the aid of an ANN to forecast performance degradation optimizing the operating conditions [45]. Farsi and Rosen performed a multi-objective optimization case study based on an ANN for a geothermal desalination system to evaluate the trade-off between exergy efficiency and process cost [46]. Nazif et al. optimized the operation of a RO system to reduce fouling, increase membrane life span, and minimize system cost using an ANN [47]. Soleimani et al. employed an ANN to derive Pareto-optimal solutions for maximizing the permeate output and minimizing the fouling resistance for membrane separation of wastewater [48].

To generate sustainable solutions for RO systems it is vital to consider the system implications to resources other than water, including energy and food, referred to as food-energy-water nexus (FEWN) [49,50,51,52] when optimizing the system. Namany et al. optimized the FEWN for various food security scenarios realizing that the utilization of RO reduces the environmental impact of solutions [53]. Tsolas et al. investigated a network representation-based graphical approach to the energy-water nexus incorporating reverse osmosis desalination systems [54]. Apart from that, Elmaadawy et al. designed a renewable energy system to meet the electrical load demand of a large-scale reverse osmosis desalination plant [55].

In this work, we employ a feed-forward ANN to approximate the energy consumption of an industrial-scale RO plant. We use hourly measured industrial-scale RO plant data, representing one and a half years of plant operation, to train and analyze several ANN set-ups and other surrogate models to comprehensively describe the complete plant behavior. A multi-objective optimization study is employed, where sustainability considerations are systematically taken into account to evaluate the trade-off between permeate production and energy consumption.

The remainder of this paper is structured as follows: The next section introduces the RO desalination plant under investigation, describes the obtained data and states the problem definition. In Section 3, all surrogate models are presented, and various ANN set-ups evaluated. Then, in Section 4, the optimization model is derived using a mixed-integer linear reformulation of all developed neural networks. Subsequently, the optimization model is used for minimizing the RO plant’s energy consumption, as well as for multi-objective optimization to analyze the trade-offs between energy consumption and purified water production. Lastly, Section 5 concludes this work.

## 2. RO Plant Description and Problem Definition

**Figure 1:**
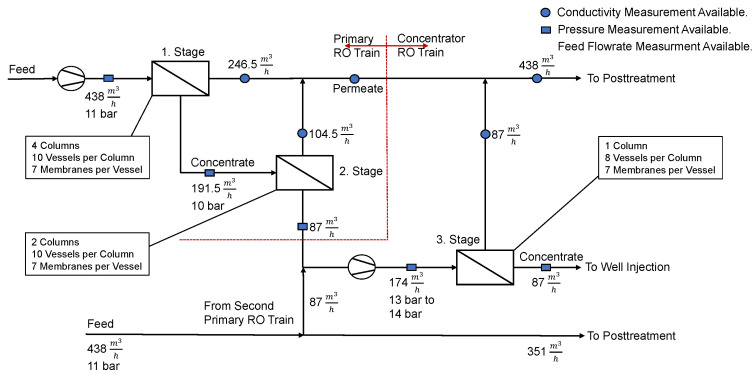
RO process overview and available measurements of the H${}_{2}$Oaks Desalination plant.

The San Antonio Water System (SAWS) H${}_{2}$Oaks Desalination plant is located in Elmendorf, Texas, just South of San Antonio, Texas. It draws water from the lower Carrizo-Wilkox Aquifer, which has a total dissolved solids (TDS) concentration of approximately $1300\;\frac{\mathrm{mg}}{L}$ [56]. The inorganic composition of the feed water is provided in Table A1 of the Appendix A. The RO system consists of three stages, four primary RO trains and two secondary or concentrator RO trains. The first and second stage build the primary RO train, whereas the third stages can be summarized as the concentrator RO train. The maximum water recovery of the first stage is $WR_{1}^{max}=56.25\%$, of the second stage is $WR_{2}^{max}=54.32\%$ and of the third stage is $WR_{3}^{max}=50\%$. Therefore the primary RO train has a maximum water recovery of $WR_{prim}^{max}=80\%$. Overall, this results in a RO plant with a maximum achievable water recovery of $WR_{sys}^{max}=90\%$. The detailed RO process flow structure is given in Figure 1. It can be seen that the permeate of two primary RO trains are blended and used as a feed for the third stage or concentrator RO train. Only pressurization of the initial feed flow and repressurization of the feed flow of the third stage are taking place. Further, the Advanced Turbo Turbocharger AT-1500 of Energy Recovery Inc. is used as an energy recovery device (ERD) in the desalination plant. It is located on the concentrate side of the third stage. The membrane system of each stage is specified in Figure 1. Throughout the plant spiral wound membranes BW30-400/34 produced by Dow Filmtec are used. All available measurement points and their respective type throughout the RO plant are given in the same figure.

**Figure 2:**
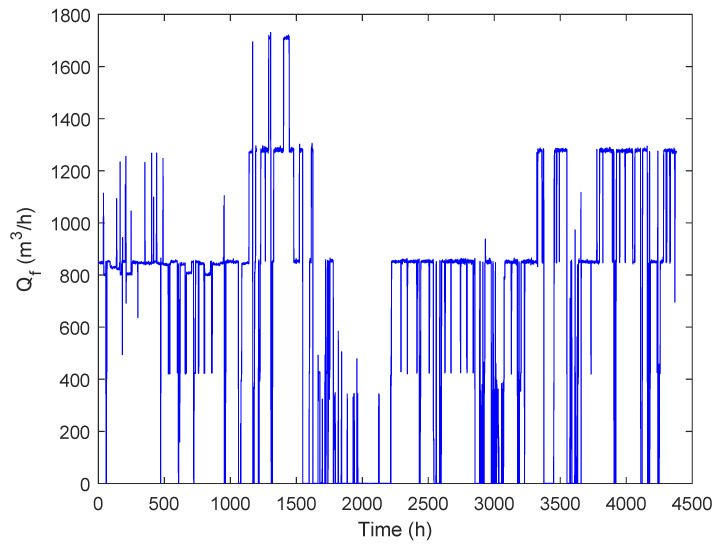
Feed flows of the RO plant of the fist six month of the year 2017.

Measurements of all marked points for one and a half years in an hourly frequency were available (beginning of 2017 to mid 2018), resulting in 14,542 data points for each parameter. In January 2017, the H${}_{2}$Oaks desalination plant has commenced operation, and therefore a variety of operating points are captured from 2017 to mid 2018, since the plant operation was still being tested.The various operational feed flows (e.g., zero feed flow for maintenance) being tested are presented in Figure 2. Information regarding the maximum ramp up and ramp down rates was also collected from this set of data, with the maximum rump up in one hour in 2017 being 97% or $1694.35\;\frac{m^{3}}{h}$, and the maximum ramp down in one hour being 49% or $858.53\;\frac{m^{3}}{h}$.

The concentration of the permeate flows of each stage, the permeate flow of the primary RO train and the overall permeate concentration have been measured. The first and second stage consist of four parallel RO flows, whereas the third stage consists of two parallel flows. For each respective parallel RO flow separate pressure measurements are available. Taking into account the number of membranes, columns and pressure vessels incorporated in each stage, this results roughly in a “4-2-1” RO separation set-up. Only data regarding the feed flow of the system has been collected. It is important to note that the pressure measurement of the retentate in the third stage is taking place after the energy recovery device. Also, the retentate flow of the first stage is the feed flow of the second stage. Table 1 summarizes all available data. The feed and retentate pressure of the second stage, third parallel flow, are for example given as $P_{f,2,3}$ and $P_{r,2,3}$. The permeate concentration is specified as $C_{p,stage}$ and the feed flow as $Q_{f}$. In addition, measurements regarding the concentration of the permeate after the primary RO train ($C_{p,prim,1}$, $C_{p,prim,2}$, $C_{p,prim,3}$, $C_{p,prim,4}$), as well as measurements of the overall permeate concentration of the system ($C_{p,sum}$) are available. It is worth noting that conductivity ($\frac{\mu S}{\mathrm{cm}}$) is a measure of concentration ($\frac{\mathrm{mg}}{L}$) and can be expressed as such [57], thus the two terms are used interchangeably throughout this work.

Thus, in this work, we present a framework for the optimal operation of industrial-scale RO desalination plants, using plant data to derive optimized process parameter results, with the goal of minimizing the overall energy consumption of the RO system. To do so, the obtained data is analyzed to derive linear surrogate models for all possible process parameters. If the dependencies between parameters are non-linear, ANNs with rectified linear units (ReLU) as activation functions are introduced to capture the observed behavior adequately. Then, the derived surrogate models can be reformulated as one mixed-integer linear programming (MILP) problem to optimize the energy consumption of the plant. The optimization model can further be modified to obtain Pareto-optimal solutions regarding the energy consumption minimization and the output water maximization. This work further includes a comparison of various ANN set-ups with changing inputs and outputs for calculating the overall permeate concentration of the plant. Depending on the research task at hand, researchers can choose models most tailored to their application.

## 3. Surrogate Modeling

Using the phenomenological transport equations for RO membranes to derive operational desalination plant parameters, results in a complex system of equations, which is computationally expensive to solve, especially within the context of optimization [24,25]. To overcome these drawbacks, linear surrogate models are employed to capture the operational RO plant behavior. To be more precise, linear correlations for the approximation of the retentate pressures of each stage and parallel flows have been derived, together with estimations of the water recovery of each stage. Then, the data has been used for the training of a feed-forward ANN with ReLUs as activation functions to approximate the permeate concentrations of each stage with the aim of calculating the overall permeate concentration of the system. An ANN with ReLUs has been selected since it can be exactly reformulated as a MILP, facilitating the optimization of the system, as it can be embedded into optimization formulations and conserve their linearity [58]. For each ANN training the obtained data has been split at random into a training set (70%), a validation set (15%) and a testing set (15%) to reduce the possibility of over- and under-fitting [59,60]. To do so, initially, the ANN is fit to the training data set by adjusting the weights and biases of all neurons in each hidden layer using the Levenberg-Marquardt algorithm to minimize the sum of squared errors [61,62]. Then, the fitted model is used for an unbiased evaluation of a second data set called validation, while the model’s hyperparameters are tuned. Lastly, the final model is used for an unbiased approximation of the independent test data set [63]. Further, for all following surrogate models each data set has been normalized between −1 and 1. All reported root mean square error (R) values are referring to the normalized data and the complete data set under investigation (training, validation and testing for ANNs). Apart from that, a linearized objective function based on the energy consumption of the RO system is derived.

### 3.1. Retentate Pressures

To calculate the retentate pressure of each stage *i* and parallel flow *j* a linear correlation based on the respective feed pressure has been assumed, as summarized in Equation (1).

(1) $$P_{r,i,j}=a_{i,j}\cdot P_{f,i,j}+b_{i,j}$$

The results of the approximation are summarized in Figure 3, Figure 4 and Figure 5 for one of the parallel flows of the first, second and third stage. The remaining results can be found in the Appendix A Table A2, Table A3, Table A4 and Table A5 and Figure A1, Figure A2, Figure A3, Figure A4, Figure A5, Figure A6 and Figure A7.

**Figure 6:**
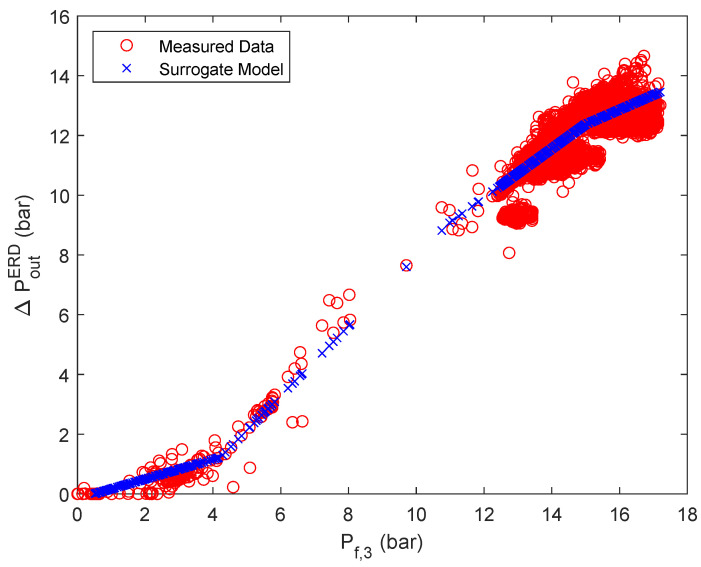
ANN with ReLUs for the approximation of the pressure difference across the ERD in stage 3.

**Figure A8:**
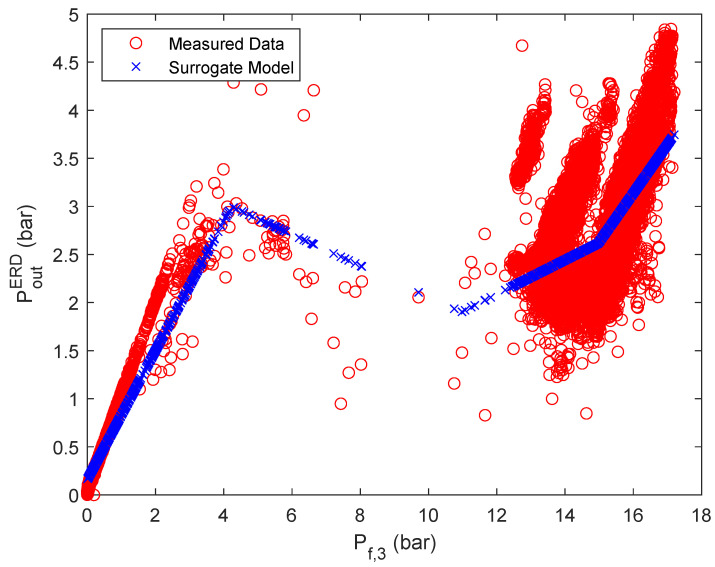
Calculation results $P_{out}^{ERD}$ based on the surrogate model for $\Delta P_{out}^{ERD}$. Both parallel flows are taken into account.

Regarding stage 3, it can be seen that even with a relatively good root mean square error of R=0.97 the actual behavior is not captured adequately. A reason for this is most likely that in this case, the pressure measurement occurs after the energy recovery device and not immediately after the RO unit. Nevertheless, for the energy optimization it is especially important to calculate the pressure drop across the energy recovery device ($\Delta P_{ERD,j},\;j=\left\{1,2\right\}$) since it can be used accordingly for energy recovery and therefore has the potential to improve the energy efficiency of the system [64]. Therefore, for the third stage, the pressure difference across the ERD is approximated with an ANN with ReLU activation functions. To calculate said pressure difference from the obtained data, it is assumed that the pressure drop across the RO unit is negligible ($P_{f,3,j}=P_{r,3,j},\;j=\left\{1,2\right\}$) for sufficiently high pressures [65]. Further, the data set for each parallel flow has been united in one overall data set, since the plant only uses one ERD. The most advantageous ANN in terms of simplicity and accuracy was obtained for one hidden layer and three nodes, resulting in an overall root mean square error of R=0.9983. Further, the derived weights and biases of the ANN are summarized in Table A6 of the Appendix A. In this case, not only the accuracy could be improved, but the surrogate model follows more closely the observed trend, as can be seen in Figure 6. However, using the obtained ANN for calculating the output pressures of the ERD based on the ANN ($P_{out,j}^{ERD}=P_{f,3,j}-\Delta P_{ERD,j}$) did not yield satisfactory results, as can be seen in Figure A8. It is important to note that $P_{r,3,j}$ as previously mentioned in Section 2, is now renamed as $P_{out,j}^{ERD}$.

**Figure 7:**
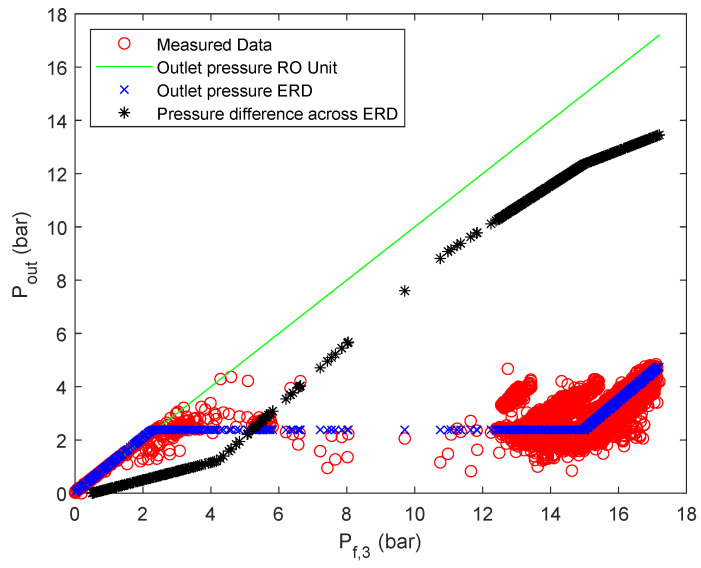
Stage 3 pressure description.

**Figure A9:**
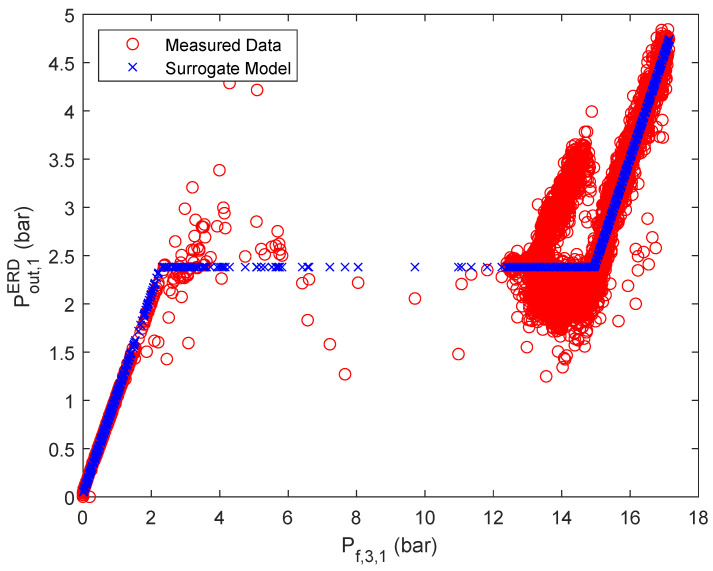
Approximation of $P_{out}^{ERD,1}$ with a separate ANN, based on stage 3 parallel flow one.

**Figure A10:**
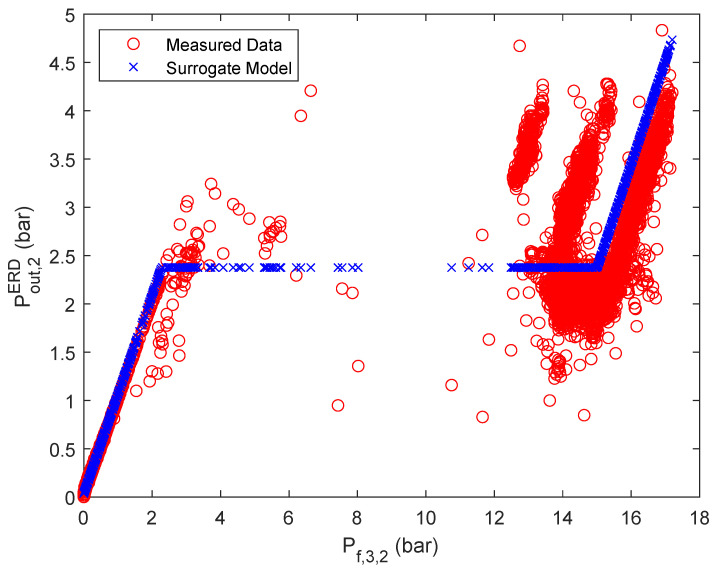
Approximation of $P_{out}^{ERD,2}$ with a separate ANN, based on stage 3 parallel flow one.

Since it may be desired to not only approximate the pressure difference across the ERD, but also the outlet pressure of the ERD, another ANN with ReLUs as activation functions is utilized to to so. The most advantageous result was obtained when the ANN was only trained with the data set of parallel flow one, resulting in R=0.9786, with one hidden layer and two nodes. The derived weights and biases of the ANN are summarized in Table A7 of the Appendix A. The results of the surrogate are summarized in Figure A9 and Figure A10. The presented approach results overall in a mathematical description of the pressures of stage 3 according to Figure 7.

### 3.2. Water Recoveries

To ensure that the RO plant operates adequately in terms of water consumption, the water recoveries have been estimated for each stage based on the actual pressure measured $P_{i,j}$ and the maximum observed pressure $max(P_{i,j})$ in each stage and parallel flow. A linear correlation between the pressures of each parallel flow and the water recovery of the respective stage is assumed [66], as introduced in Equation (2). Then, based on an overall mass balance the RO plant water recovery can be calculated according to Equation (3).

(2) $$WR_{i}=WR_{i}^{max}\cdot \frac{\sum_{j}P_{i,j}}{\sum_{j}max\left(P_{i,j}\right)}$$

(3) $$WR_{sys}=WR_{1}+WR_{2}\cdot \left(1-WR_{1}\right)+WR_{3}\cdot \left(1-WR_{2}\right)\cdot \left(1-WR_{1}\right)$$

This approach results in water recovery estimations based on the obtained data as specified in Table 2.

### 3.3. Permeate Concentration

Since surrogate models for the retentate pressures and energy recovery have already been defined, the only variable left to compute is the overall permeate concentration. To do so, again an ANN with ReLUs as activation functions is employed. Further, instead of simply using defined inputs as the sole inputs of the ANN (input={in(t)}) to compute the outputs (output={out(t)}), it is also possible to consider as an additional input the inputs of the previous time point (input={in(t),in(t−1)}) or the outputs of the previous time point (input={in(t),out(t−1)}). The latter two approaches are used to capture the dynamics of the system. For all here presented ANN results, the last approach (input={in(t),out(t−1)}) was selected, since the R value was the highest for all investigated cases, compared to the other two set-ups.

Generally, with the available data, it is possible to generate surrogate models based on ANNs for several plant set-ups. It is possible to generate an ANN for each respective stage, for each RO train or for the complete RO plant. Further, several different process parameters can be considered as inputs of the ANN, as summarized in Table 3. It is worthy to note that adding several layers has been investigated for all presented cases as well. However, the accuracy could not be improved. The presented number of nodes (#nodes) in the hidden layer represents the number of nodes after which no significant change in the R value (second decimal) could be detected by further increasing the number of nodes.

**Figure A11:**
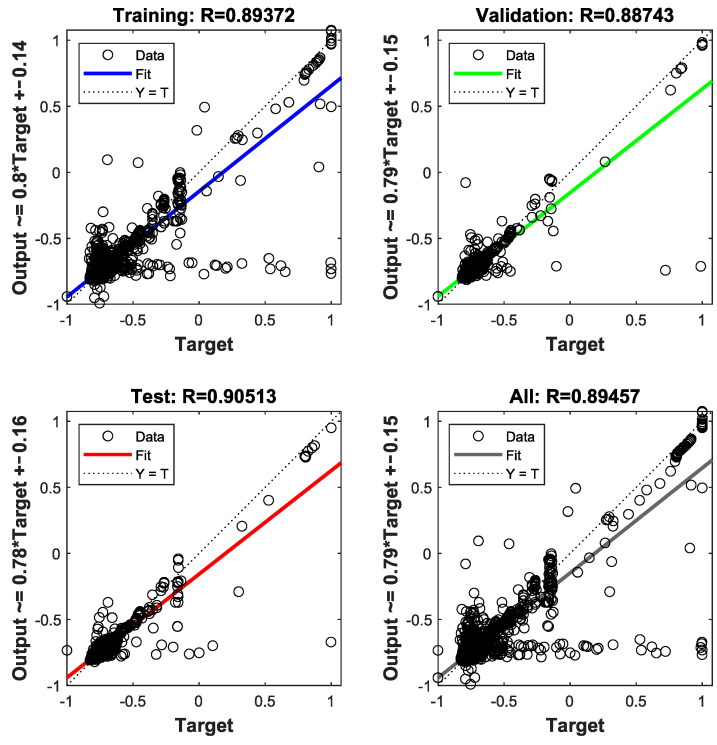
Results of the ANN training for the approximation of the overall permeate concentration, R = 0.895.

Moreover, Table 3 also summarizes the results of each ANN training in terms of R, to compare the accuracy of the derived surrogate models. In this case, we desire a compromise between accuracy and simplicity of the model. The most accurate networks are being generated, when each stage is modeled separately. However, in this case the results of each surrogate have to be used for further calculations, i.e., closing the overall permeate mass balance, which reduces the accuracy of the obtained results in terms of approximating the overall permeate concentration of the system. In addition, more nodes are required to achieve these high R values (with the expectation of stage 3). Apart from that, in only 56 out of 14,543 data points (0.39%) the observed permeate concentration is higher than the restriction for drinking water ($500\;\frac{\mathrm{mg}}{L}$ [67]), with a maximum observed overall permeate concentration of $626\;\frac{\mathrm{mg}}{L}$. Therefore, we decided to use the overall RO plant surrogate model one (R = 0.895), which is arguably one of the least accurate surrogates of the presented ones, but by far the simplest one and yet still sufficiently accurate for the purpose of energy optimization, while fulfilling the drinking water quality restriction. The results of the ANN training for the selected case, split into the training, testing, validation, as well as showing the overall result, is summarized in Figure A11 of the Appendix A. Additionally, the derived weights and biases of the ANN are presented in Table A8 of the Appendix A.

**Figure A12:**
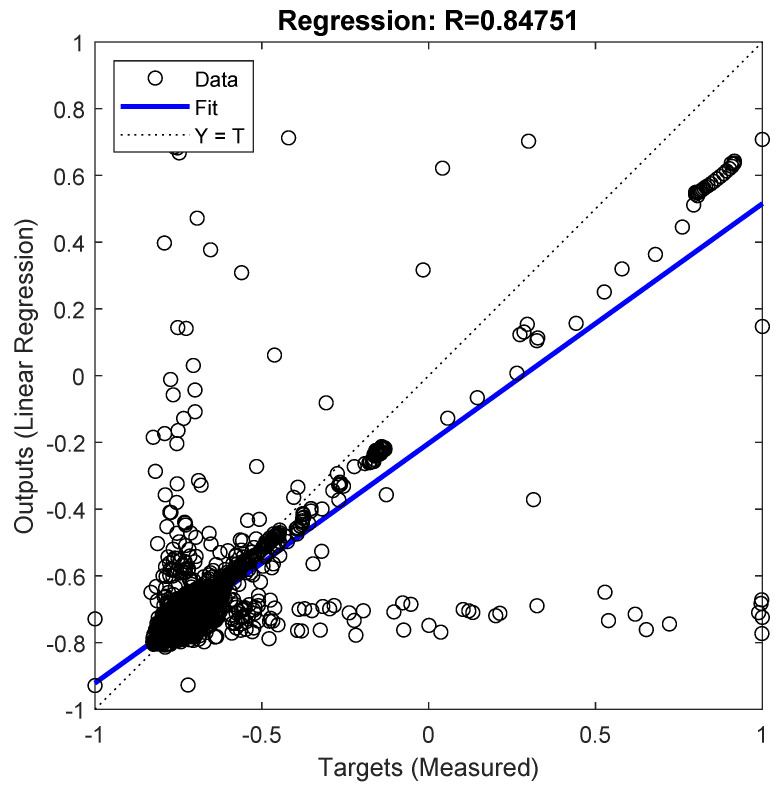
Normalized results multivariate linear regression of the RO system to approximate the overall permeate concentration $C_{p,sum}$, R = 0.848.

It is important to mention that also a multivariate linear regression was employed instead of using an ANN, for the same inputs and outputs as the selected surrogate, which resulted in less accurate approximations (R = 0.848), as can be seen in Figure A12.

### 3.4. Objective Function

To calculate the overall RO plant energy consumption, the specific energy consumption (SEC) of a single pump, according to Equation (4), is applied to the RO system, resulting in an expression as shown in Equation (5) [68].

(4) $$SEC=\frac{Q_{f}\cdot \Delta P}{\eta \cdot Q_{p}}$$

(5) $$\begin{array}{cc} SEC & =\frac{Q_{f}}{Q_{p}}\cdot (\frac{1}{4\cdot \eta_{1}}\sum_{j=1}^{i=4}P_{f,1,j}+\frac{\left(1-WR_{1}\right)\left(1-WR_{2}\right)}{\eta_{1}}\left(\frac{1}{2}\sum_{j=1}^{i=2}\cdot P_{f,3,j}-\frac{1}{4}\sum_{j=1}^{i=4}P_{r,2,j}\right)-\cdots \\ \; & \cdots \frac{\left(1-WR_{1}\right)\left(1-WR_{2}\right)\left(1-WR_{3}\right)\cdot \eta_{2}}{2}\sum_{j=1}^{i=2}\Delta P_{ERD,j}) \end{array}$$

Here, $\eta_{1}$ denotes the pump efficiency and is assumed to be 0.74. In contrast, the efficiency of the ERD is assumed to be $\eta_{2}=0.8$ [69]. The remaining factors are derived from mass balance equations considering the process flows, as presented in Figure 1. To obtain a linear objective function $WR_{1}$, $WR_{2}$ and $WR_{3}$ are substituted for their respective maximum possible values and the first factor is neglected, resulting in Equation (6):

(6) $$\begin{array}{cc} SEC^{*} & =\frac{1}{4\cdot \eta_{1}}\sum_{j=1}^{i=4}P_{f,1,j}+\frac{1}{10\cdot \eta_{1}}\left(\sum_{j=1}^{i=2}P_{f,3,j}-\frac{1}{2}\sum_{j=1}^{i=4}P_{r,2,j}\right)-\frac{\eta_{2}}{20}\sum_{j=1}^{i=2}\Delta P_{ERD,j} \end{array}$$

## 4. Optimization Model

To incorporate the ANN to calculate the pressure difference across the ERD and the ANN to approximate the permeate concentration in the optimization model, both ANN models are reformulated as MILPs, as presented in [58,70]. Therefore, the weights and biases of the former ANN are referred to as $W_{k,l}^{ERD}$ and $b_{k,l}^{ERD}$, where the hidden and output layer can be distinguished with k={1,2} and the number of nodes in the hidden layer are given as l={1,2,3}. For the latter ANN, an additional index h={1,2,3,4,5} has to be introduced to distinguish the weights of the input layer for the set of inputs, resulting in $W_{h,k,l}^{RO}$ and $b_{k,l}^{RO}$. Further, auxiliary variables $x_{1,l,j}$, $s_{1,l,j}$ and $z_{1,l,j}$ are introduced for the reformulation of the ANN approximating $\Delta P_{ERD,j}$. Accordingly, for the ANN calculating $C_{p,t}$, $x_{2,l}$, $s_{2,l}$ and $z_{2,l}$ are introduced. To differentiate between the permeate concentration of a previous time point and the permeate concentration in the time point under investigation the indices *t* and t−1 are utilized. Together with the other surrogate models for the retentate pressures and the water recovery estimation, this results in the following optimization model (Equations (7)–(25)) to minimize the overall energy consumption of the RO system.

(7) $$\min\;SEC^{*}=\frac{1}{4\cdot \eta_{1}}\sum_{j=1}^{i=4}P_{f,1,j}+\frac{1}{10\cdot \eta_{1}}\left(\sum_{j=1}^{i=2}P_{f,3,j}-\frac{1}{2}\sum_{j=1}^{i=4}P_{r,2,j}\right)-\frac{\eta_{2}}{20}\sum_{j=1}^{i=2}\Delta P_{ERD,j}$$

(8) $$s.t.\;Q_{f}\geq Q_{f}^{res}$$

(9) $$WR_{i}\geq WR_{i}^{res},\;\forall i=\left\{1,2,3\right\}$$

(10) $$C_{p,t}\leq C_{p}^{res}$$

(11) $$C_{p,t}=C_{p,t-1}$$

(12) $$WR_{i}=WR_{i}^{max}\cdot \frac{\sum_{j}P_{i,j}}{\sum_{j}max\left(P_{i,j}\right)},\;\forall i=\left\{1,2,3\right\}$$

(13) $$P_{r,i,j}=a_{i,j}\cdot P_{f,i,j}+b_{i,j},\;\forall i=\left\{1,2\right\},j=\left\{1,2,3,4\right\}$$

(14) $$P_{f,2,j}=P_{r,1,j},\;\forall j=\left\{1,2,3,4\right\}$$

(15) $$W_{1,l}^{ERD}\cdot P_{f,3,j}+b_{1,l}^{ERD}=x_{1,l,j}-s_{1,l,j},\;\forall j=\left\{1,2\right\},l=\left\{1,2,3\right\}$$

(16) $$\sum_{l=1}^{3}W_{2,l}^{ERD}\cdot x_{1,l,j}+b_{2}^{ERD}=\Delta P_{ERD,j},\;\forall j=\left\{1,2\right\}$$

(17) $$x_{1,l,j}-z_{1,l,j}\cdot U_{1,l,j}\leq 0,\;\forall j=\left\{1,2\right\},l=\left\{1,2,3\right\}$$

(18) $$s_{1,l,j}-\left(1-z_{1,l,j}\right)\cdot L_{1,l,j}\leq 0,\;\forall j=\left\{1,2\right\},l=\left\{1,2,3\right\}$$

(19) $$W_{1,1,l}^{RO}\cdot Q_{f}+\sum_{i=1}^{3}W_{1,i+1,l}^{RO}\cdot WR_{i}+W_{1,5,l}^{RO}\cdot C_{p,t-1}+b_{1,l}^{ERD}=\cdots$$

(20) $$\cdots =x_{2,l}-s_{2,l},\;\forall l=\left\{1,2,3\right\}$$

(21) $$\sum_{l=1}^{3}W_{2,l}^{RO}\cdot x_{2,l}+b_{2}^{ERD}=C_{p,t}$$

(22) $$x_{2,l}-z_{2,l}\cdot U_{2,l}\leq 0,\;\forall l=\left\{1,2,3\right\}$$

(23) $$s_{2,l}-\left(1-z_{2,l}\right)\cdot L_{2,l}\leq 0,\;\forall l=\left\{1,2,3\right\}$$

(24) $$x_{1,l,j},s_{1,l,j},x_{2,l},s_{2,l}\geq 0,\;\forall l=\left\{1,2,3\right\},j=\left\{1,2\right\}$$

(25) $$z_{1,l,j},z_{2,l}\in \left\{0,1\right\},\;\forall l=\left\{1,2,3\right\},j=\left\{1,2\right\}$$

The objective function denoting the energy consumption of the RO plant, as derived in the previous section, is presented in Equation (7). To calculate the overall energy consumption in kW, the objective function is multiplied with $Q_{f}$. To derive the specific energy consumption in $\frac{\mathrm{kWh}}{m^{3}}$, the objective is divided by the overall water recovery of the system as calculated with Equation (3). From Equations (8)–(10) process restrictions are introduced to guarantee that a minimum feed flow and a minimum water recovery per stage are fulfilled, to produce drinking water ($C_{p}^{res}=500\;\frac{\mathrm{mg}}{L}$). Equation (11) is introduced to incorporate a steady-state process assumption and guarantee that in t−1 the water quality is comparable to the one obtained in *t*. Then, the water recoveries of each stage based on the presented linear pressure correlations (see Equation (2)) of the pressure in the respective parallel flows of each stage (Equation (12)) and the retentate pressure of stages one and two according to the linear pressure correlations are calculated (Equation (13)). Further, the retentate pressure of stage one is used as the feed pressure of stage two (Equation (14)). Equations (15)–(18) summarize the MILP reformulation of the ANN to approximate the pressure difference across the ERD, whereas Equations (20)–(23) are used as the representation of the ANN to calculate the overall permeate concentration. To successfully implement these reformulations, the auxiliary variables $x_{1,l,j},s_{1,l,j},x_{2,l}$ and $s_{2,l}$ have to be positive, to split the ReLU output into a positive and negative component according to the binary variables $z_{1,l,j}$ and $z_{2,l}$ (Equations (24) and (25)). This ReLU output distinction is further implemented with the inequality constraints shown in Equations (17), (18), (22) and (23), which introduce lower and upper boundaries for each output of a node in the hidden layer ($L_{1,l,j},U_{1,l,j}$ and $L_{2,l},U_{2,l}$). Overall, the optimization model results in a MILP, which was solved in MATLAB using the CPLEX solver.

## 5. Results and Discussion

Next, the optimization model is used for two studies. First of all, the energy of the system is minimized for distinct water recovery and feed flow restrictions to enable a comparison of generated results with the actual energy consumption of the desalination plant. Then, the trade-off between energy minimization and water utilization in terms of the feed flow and the overall water recovery are visualized in a Pareto front. To do so, the ϵ-constrained method is used for multi-objective optimization [71].

### 5.1. Energy Minimization

To evaluate the optimization model, the overall energy consumption of the RO plant for the year 2017 is compared to the energy optimization results. Therefore, the data set for the year 2017 for the feed flow and the estimated water recovery of the system are split into 12 sets according to the month of the year. Then, the monthly average of the feed flow and the overall water recovery are calculated. The obtained values are summarized in Table 4. These values are used as a pairwise input of the optimization model to update the restrictions shown in Equations (8) and (9). More specifically, the optimization model is solved while the restrictions of Equation (9) are updated to fulfill the set overall water recovery as presented in Table 4, calculated with Equation (3).

**Figure 8:**
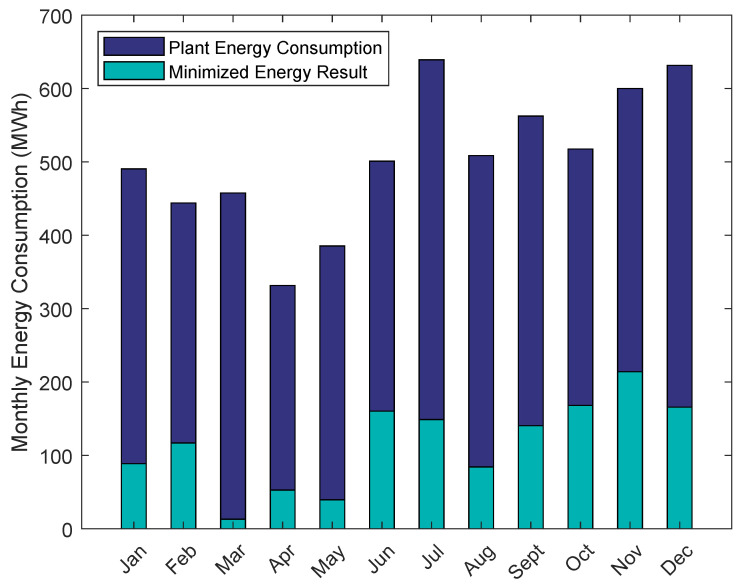
Energy minimization results of the year 2017. Comparison of minimized energy results and the overall RO plant energy consumption.

The results of this case study are presented in Figure 8. In June 2017, the fraction of the derived RO desalination energy consumption to the overall energy consumption of the plant is at a year high of 51%, to compare, in March 2017 said fraction is at a year low of 10%, whereas calculating the average results in a fraction of only 40%. Generally, the energy consumption of the RO system is the major energy consumer of a RO plant having a major impact on the process performance and sustainability [72]. Therefore, the minimized energy consumption of the RO system is comparably low, i.e., less than half the overall consumed energy of the plant on average, yielding satisfactory results which can lead to substantial energy savings.

Originally, the obtained monthly energy consumptions were planned to be compared to literature values. However, the energy consumption of RO plants is reported in the literature normalized with the permeate output in $\frac{\mathrm{kWh}}{m^{3}\;\mathrm{permeate}}$. The here presented results are derived in MWh. However, the SEC of the plant for various operating points is presented and compared to literature values in Section 5.2. For now, the results of the energy optimization are compared to the overall energy consumption of the high service pumps of the desalination plant, to classify the order of magnitude of results. In 2017, the high service pumps consumed $1.5169\times 10^{3}\;\frac{\mathrm{MWh}}{\mathrm{year}}$. Summing up the obtained results to calculate the energy consumption for 2017 results in $1.3929\times 10^{3}\;\frac{\mathrm{MWh}}{\mathrm{year}}$. Consequently, the difference in energy consumption is 124 MWh or around 8%. Since the overall energy consumption of the RO system is in the same order of magnitude as the energy consumption of the high service pumps, the initial statement of obtaining advantageous results, in terms of minimizing the systems energy consumption, is confirmed.

### 5.2. Multi-Objective Optimization

**Figure 9:**
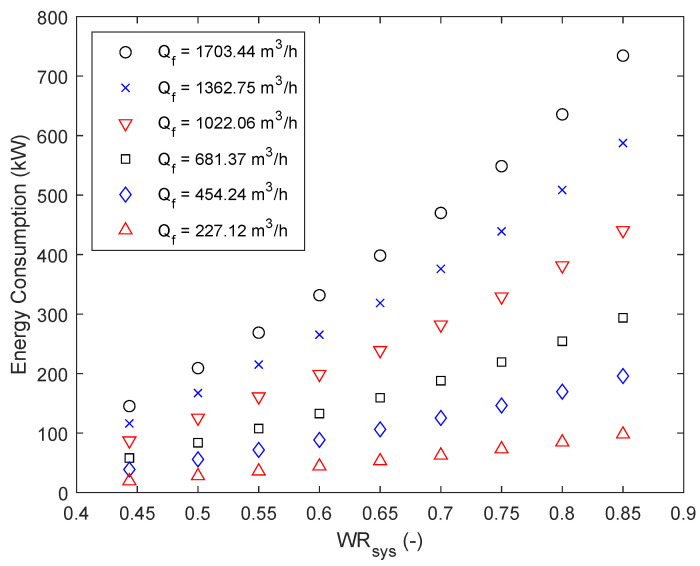
Pareto front evaluating the trade-off between minimizing the energy consumption in kW, the feed flow, as well as the water recovery of the system.

After confirming the capabilities of the optimization model, the model was expanded to consider multiple objectives using multi-objective optimization. To do so, the constraints summarized in Equations (8) and (9) are updated to calculate the minimized energy for a set of water recoveries and feed flows. Compared to the previous study, where a pair of water recovery and feed flow were the inputs, an array of water recoveries and feed flows are used as inputs, performing the minimization at each feed flow for all specified water recoveries. The multi-objective optimization has only been performed in the RO plant’s relevant water recovery region of 40% to 85%. The obtained Pareto-optimal solutions are visualized in Figure 9. It can be seen that the energy consumption increases with increasing water recoveries, as well as increasing feed flows. Apart from that, it can be deducted that the energy consumption from the lowest water recovery to the highest water recovery for all feed flows increases approximately by a factor of 5. Additionally, comparing the calculated energy consumption for the highest feed flow with the lowest one, results in the deduction that the gap between the two feed flows increases with increasing water recoveries. For a water recovery of 50% the energy consumption difference between the two is approximately 125,883 kW, whereas the difference increases to 636,471 kW for a water recovery of 85%. Therefore, depending on the separation task at hand, this result can lead to indirect energy savings.

To further analyze the results, the specific energy consumption in $\frac{\mathrm{kWh}}{m^{3}}$ of the results is calculated and summarized in Table 5. The specific energy consumption can be approximated with the linear correlation presented in Equation (26), with an accuracy of R = 0.998.

(26) $$SEC\;\left(\frac{\mathrm{kWh}}{m^{3}}\right)=0.753\cdot \frac{WR_{sys}\left(\%\right)}{100}-0.1326$$

As expected, the specific energy consumption increases with the water recovery of the system. Increasing the water recovery by a factor of 1.7 from 50% to 85% results in approximately doubling the specific energy consumption (factor of 2.07), from $0.2455\;\frac{\mathrm{kWh}}{m^{3}}$ to $0.5072\;\frac{\mathrm{kWh}}{m^{3}}$. This underlines the sensitivity of the specific energy consumption to changes in water recovery since the relative change between the respective factors is more than 20%. Lastly, we compared the obtained minimum specific energy consumption with literature values. Stillwell and Webber report observed literature values for the SEC of BWRO desalination plants between $0.5\;\frac{\mathrm{kWh}}{m^{3}}$ and $3\;\frac{\mathrm{kWh}}{m^{3}}$ [73]. In addition, Sassi and Mujtaba report a minimized SEC between $0.578\;\frac{\mathrm{kWh}}{m^{3}}$ and $0.730\;\frac{\mathrm{kWh}}{m^{3}}$ [20]. The highest here observed SEC is $0.5072\;\frac{\mathrm{kWh}}{m^{3}}$ for $WR_{sys}=85\%$. Overall, this comparison underlines the competitiveness of the presented optimization methodology, as well as the potential energy savings when this approach is employed.

## 6. Conclusions

We presented a comprehensive methodology to utilize industrial scale RO desalination plant data for surrogate modeling and subsequent optimization. Linear surrogate models were developed for the retentate pressures of the parallel flows of the first and second stage. For the pressure difference across the ERD, as well as the output pressure of the ERD, ANNs with ReLUs were trained and reformulated as MILPs with the feed pressure of stage three as the sole input. To calculate the permeate concentration of the system several possible ANN formulations have been presented and compared in terms of their respective inputs and accuracy (R). Depending on the task at hand different surrogate models can be selected. For the subsequent optimization case studies the simplest ANN system resulted in sufficiently accurate results and was therefore reformulated as a MILP.

The optimization model was first used for minimizing the energy consumption of the RO plant based on the monthly averaged feed flows and water recoveries of the year 2017. The derived energy consumption constituted at most 51% of the monthly overall RO desalination energy consumption and on average 40% throughout the year, underlining the major energy saving potential using the presented methodology. In fact, the derived energy consumption is on the same order of magnitude as the energy consumption of the high service pumps of the RO plant.

Then, the model was used to derive a Pareto front to illustrate the trade-offs between minimizing the energy of the system as well as maximizing the feed flow and water recovery of the system. Here, the potential for indirect savings was uncovered by comparing the increase in energy consumption for the lowest and highest utilized feed flows. The results were further analyzed in terms of the specific energy consumption of the system, which ultimately showed the significant impact of changing water recoveries on the specific energy consumption.

Overall, the presented methodology should be understood as a recipe for other researchers on how to move from obtained RO data sets to an optimization model for single and multi-objective optimization using linear data driven surrogate models. In subsequent work, the objective function can be re-evaluated by introducing a nonlinear function and compare obtained results to the linear case. Moreover, if other data points across the plant can be obtained, i.e., the permeate flows or water recoveries for each stage, the accuracy of the surrogates can be further validated and if necessary modified.

## Figures and Tables

**Figure 3:**
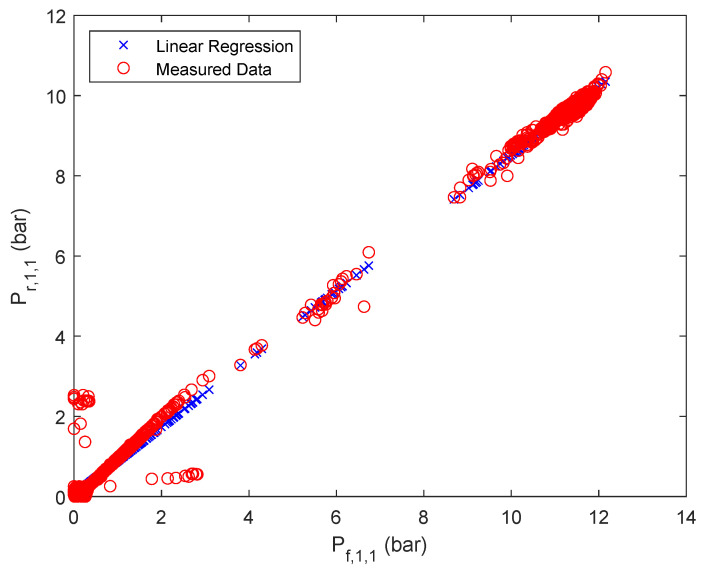
Linear regression results of retentate pressure of stage 1, first parallel flow.

**Figure 4:**
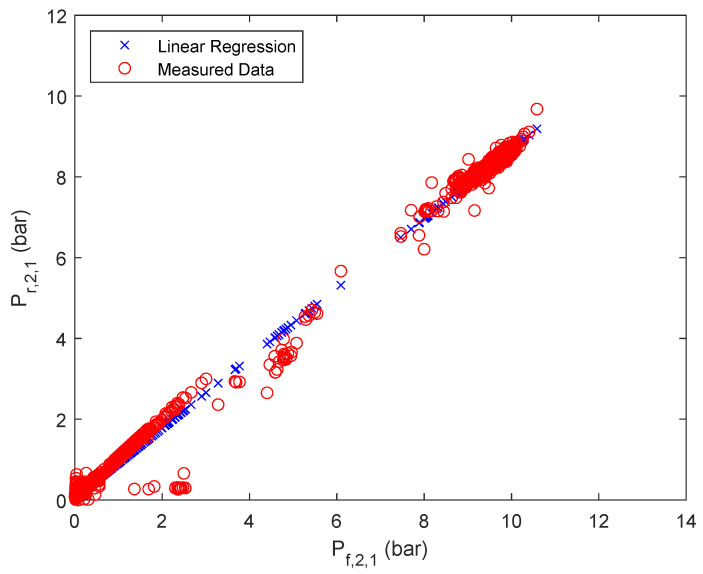
Linear regression results of retentate pressure of stage 2, first parallel flow.

**Figure 5:**
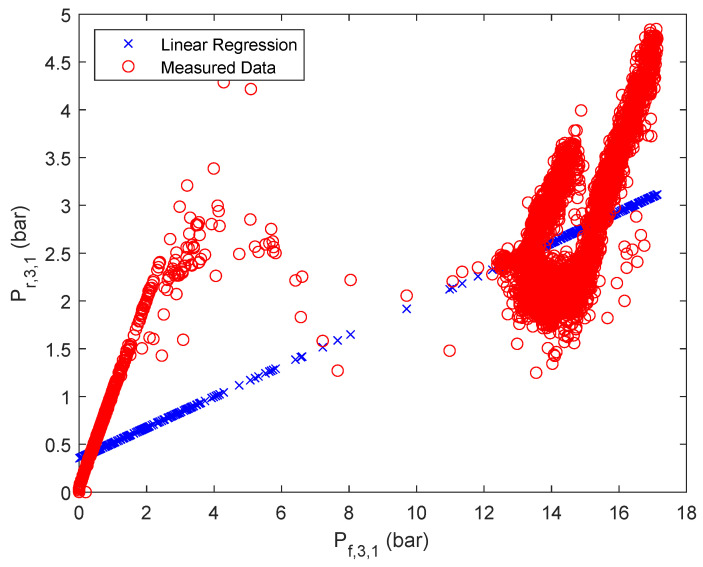
Linear regression results of retentate pressure of stage 3, first parallel flow.

**Table 1:** Overview of measured data throughout the RO plant per stage. The pressures were measured in psi and the feed flow in $\frac{\mathrm{gal}}{\min}$. Both have been converted to SI units.

Parameter	Stage 1	Stage 2	Stage 3
Feed pressure (bar)	$P_{f,1,1}$, $P_{f,1,2}$, $P_{f,1,3}$, $P_{f,1,4}$	$P_{f,2,1}$, $P_{f,2,2}$, $P_{f,2,3}$, $P_{f,2,4}$	$P_{f,3,1}$, $P_{f,3,2}$
Retentate pressure (bar)		$P_{r,2,1}$, $P_{r,2,2}$, $P_{r,2,3}$, $P_{r,2,4}$	$P_{r,3,1}$, $P_{r,3,2}$
Permeate Conductivity ($\frac{\mu S}{\mathrm{cm}}$)	$C_{p,1,1}$, $C_{p,1,2}$, $C_{p,1,3}$, $C_{p,1,4}$	$C_{p,2,1}$, $C_{p,2,2}$, $C_{p,2,3}$, $C_{p,2,4}$	$C_{p,3,1}$, $C_{p,3,2}$
Feed flow ($\frac{m^{3}}{h}$)	$Q_{f}$		

**Table 2:** Results of estimating the water recoveries throughout the RO plant.

Parameter	Mean	Max	Min
$WR_{1}$	31.13%	52.77%	0.37%
$WR_{2}$	29.35%	50.16%	0.34%
$WR_{3}$	18.60%	39.50%	0%
$WR_{sys}$	57.92%	85.66%	0.97%

**Table 3:** Inputs and outputs of various possible ANNs. In each case only one hidden layer has been considered (input={in(t),out(t−1)}; output={out(t)} ).

Approach	Input	Output	#Nodes	*R*
	$Q_{f}$, $WR_{1}$	$C_{p,1,1}$, $C_{p,1,2}$,	7	0.961
		$C_{p,1,3}$, $C_{p,1,4}$		
	$Q_{f}$, $WR_{1}$,	$C_{p,1,1}$, $C_{p,1,2}$,	10	0.973
Stage 1	$P_{f,1,1}$, $P_{f,1,2}$, $P_{f,1,3}$, $P_{f,1,4}$	$C_{p,1,3}$, $C_{p,1,4}$		
	$Q_{f}$, $WR_{1}$,	$C_{p,1,1}$, $C_{p,1,2}$, $C_{p,1,3}$, $C_{p,1,4}$	9	0.972
	$P_{f,1,1}$, $P_{f,1,2}$, $P_{f,1,3}$, $P_{f,1,4}$,			
	$P_{f,2,1}$, $P_{f,2,2}$, $P_{f,2,3}$, $P_{f,2,4}$			
	$\left(1-WR_{1}\right)\cdot Q_{f}$, $WR_{2}$	$C_{p,2,1}$, $C_{p,2,2}$,	8	0.958
		$C_{p,2,3}$, $C_{p,2,4}$		
	$\left(1-WR_{1}\right)\cdot Q_{f}$, $WR_{2}$,	$C_{p,2,1}$, $C_{p,2,2}$,	12	0.965
Stage 2	$P_{f,2,1}$, $P_{f,2,2}$, $P_{f,2,3}$, $P_{f,2,4}$	$C_{p,2,3}$, $C_{p,2,4}$		
	$\left(1-WR_{1}\right)\cdot Q_{f}$, $WR_{2}$,	$C_{p,2,1}$, $C_{p,2,2}$, $C_{p,2,3}$, $C_{p,2,4}$	11	0.964
	$P_{f,2,1}$, $P_{f,2,2}$, $P_{f,2,3}$, $P_{f,2,4}$,			
	$P_{r,2,1}$, $P_{r,2,2}$, $P_{r,2,3}$, $P_{r,2,4}$			
	$Q_{f}$, $WR_{1}$, $WR_{2}$	$C_{p,prim,1}$, $C_{p,prim,2}$,	8	0.952
		$C_{p,prim,3}$, $C_{p,prim,4}$		
	$Q_{f}$, $WR_{1}$, $WR_{2}$,	$C_{p,prim,1}$, $C_{p,prim,2}$, $C_{p,prim,3}$, $C_{p,prim,4}$	5	0.951
	$P_{f,1,1}$, $P_{f,1,2}$, $P_{f,1,3}$, $P_{f,1,4}$,			
Primary RO train	$P_{f,2,1}$, $P_{f,2,2}$, $P_{f,2,3}$, $P_{f,2,4}$			
	$Q_{f}$, $WR_{1}$, $WR_{2}$,	$C_{p,prim,1}$, $C_{p,prim,2}$, $C_{p,prim,3}$, $C_{p,prim,4}$	6	0.954
	$P_{f,1,1}$, $P_{f,1,2}$, $P_{f,1,3}$, $P_{f,1,4}$,			
	$P_{f,2,1}$, $P_{f,2,2}$, $P_{f,2,3}$, $P_{f,2,4}$			
	$P_{r,2,1}$, $P_{r,2,2}$, $P_{r,2,3}$, $P_{r,2,4}$			
	$\left(1-WR_{1}\right)\cdot \left(1-WR_{2}\right)\cdot Q_{f}$,	$C_{p,3,1}$, $C_{p,3,2}$	3	0.954
	$WR_{3}$			
	$\left(1-WR_{1}\right)\cdot \left(1-WR_{2}\right)\cdot Q_{f}$,	$C_{p,3,1}$, $C_{p,3,2}$	5	0.968
Stage 3	$WR_{3}$, $P_{f,3,1}$, $P_{f,3,2}$			
	$\left(1-WR_{1}\right)\cdot \left(1-WR_{2}\right)\cdot Q_{f}$,	$C_{p,3,1}$, $C_{p,3,2}$	4	0.968
	$WR_{3}$, $P_{f,3,1}$, $P_{f,3,2}$			
	$P_{r,3,1}$, $P_{r,3,2}$			
	$Q_{f}$, $WR_{1}$, $WR_{2}$, $WR_{3}$,	$C_{p,sum}$	3	0.895
	$Q_{f}$, $WR_{1}$, $WR_{2}$, $WR_{3}$,	$C_{p,sum}$	4	0.894
	$P_{f,1,1}$, $P_{f,1,2}$, $P_{f,1,3}$, $P_{f,1,4}$			
	$P_{f,3,1}$, $P_{f,3,2}$			
Overall plant	$Q_{f}$, $WR_{1}$, $WR_{2}$, $WR_{3}$,	$C_{p,sum}$	3	0.898
	$P_{f,1,1}$, $P_{f,1,2}$, $P_{f,1,3}$, $P_{f,1,4}$			
	$P_{f,2,1}$, $P_{f,2,2}$, $P_{f,2,3}$, $P_{f,2,4}$			
	$P_{r,2,1}$, $P_{r,2,2}$, $P_{r,2,3}$, $P_{r,2,4}$			
	$P_{f,3,1}$, $P_{f,3,2}$, $P_{r,3,1}$, $P_{r,3,2}$			

**Table 4:** Monthly averaged feed flow and water recovery of the RO plant for the year 2017.

Parameter	Jan	Feb	Mar	Apr	May	Jun	Jul	Aug	Sept	Oct	Nov	Dec
$Q_{f}(\cdot 10^{2}\;\frac{m^{3}}{h}$)	8.11	9.52	2.13	6.95	6.45	10.7	10.2	8.29	9.86	10.6	11.7	10.7
$WR_{sys}\left(\%\right)$	54.2	56.8	14.5	47.41	40.0	61.9	60.9	52.6	60.4	63.2	67.4	62.8

**Table 5:** Specific energy consumption of the obtained multi-objective optimization results.

${\mathrm{WR}}_{\mathrm{sys}}\left(\%\right)$	$\mathrm{SEC}\;(\frac{\mathrm{kWh}}{m^{3}})$
44	0.1923
50	0.2455
55	0.2868
60	0.3245
65	0.3597
70	0.3941
75	0.4294
80	0.4665
85	0.5072

**Figure A1:**
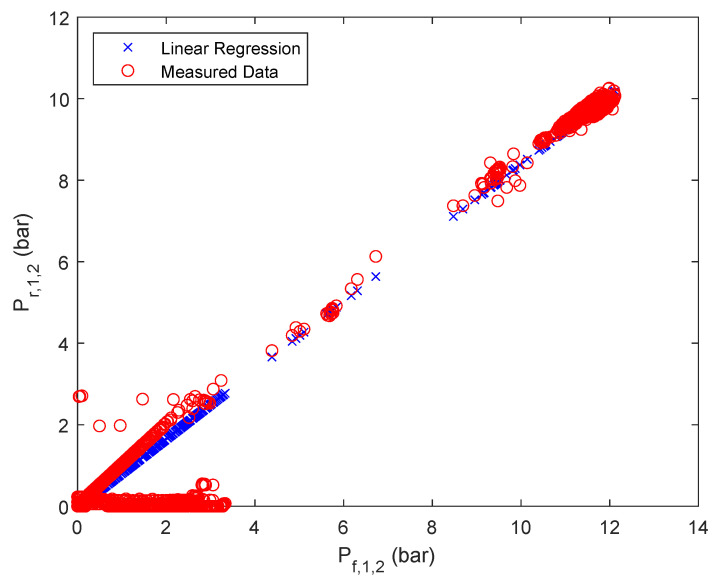
Linear regression results of retentate pressure of stage 1, second parallel flow.

**Figure A2:**
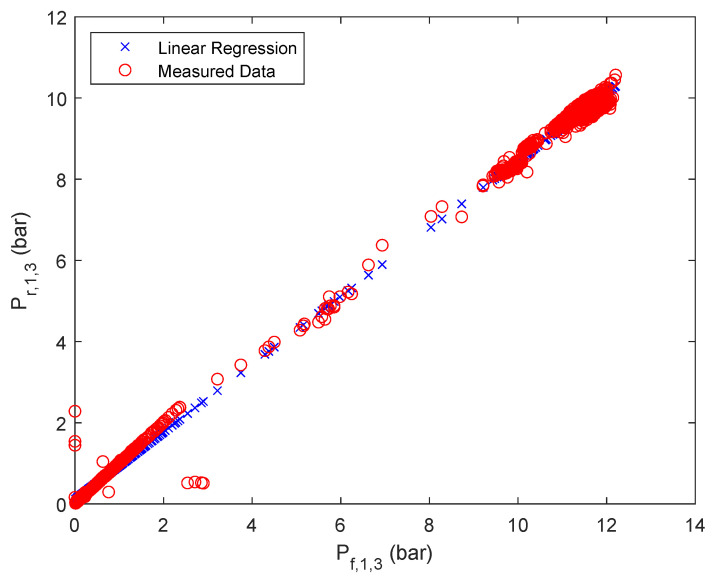
Linear regression results of retentate pressure of stage 1, third parallel flow.

**Figure A3:**
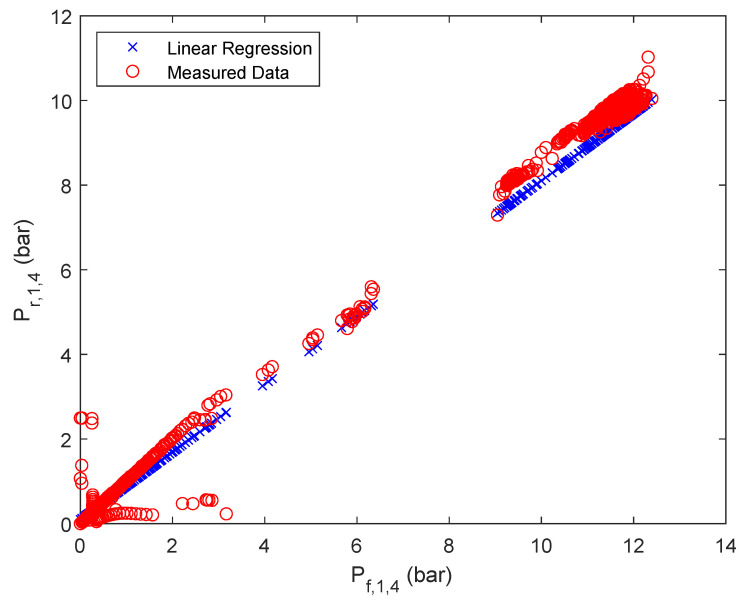
Linear regression results of retentate pressure of stage 1, fourth parallel flow.

**Figure A4:**
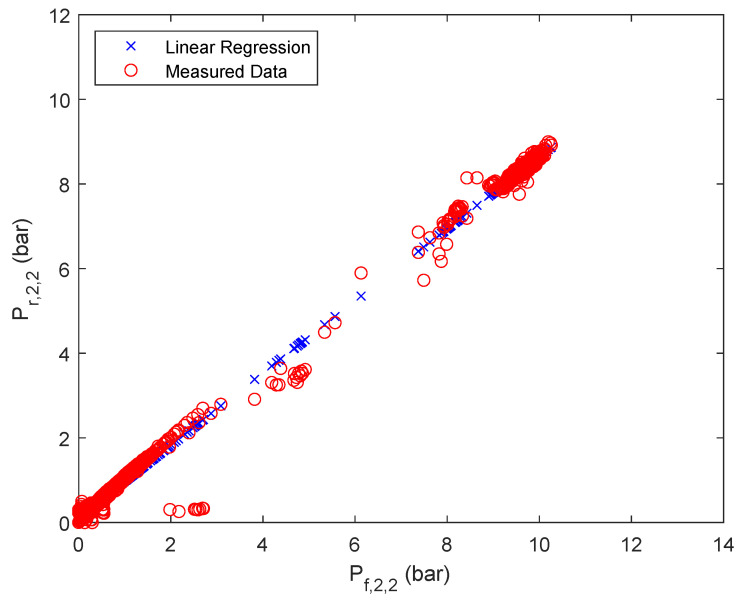
Linear regression results of retentate pressure of stage 2, second parallel flow.

**Figure A5:**
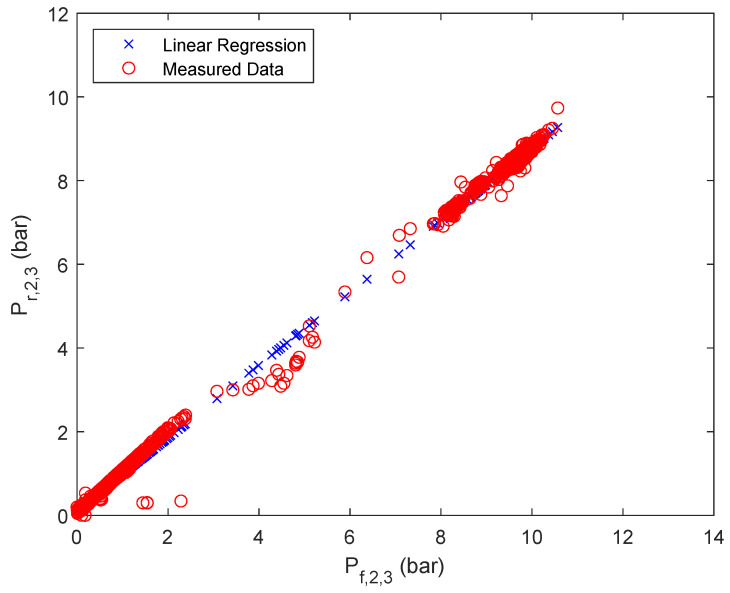
Linear regression results of retentate pressure of stage 2, third parallel flow.

**Figure A6:**
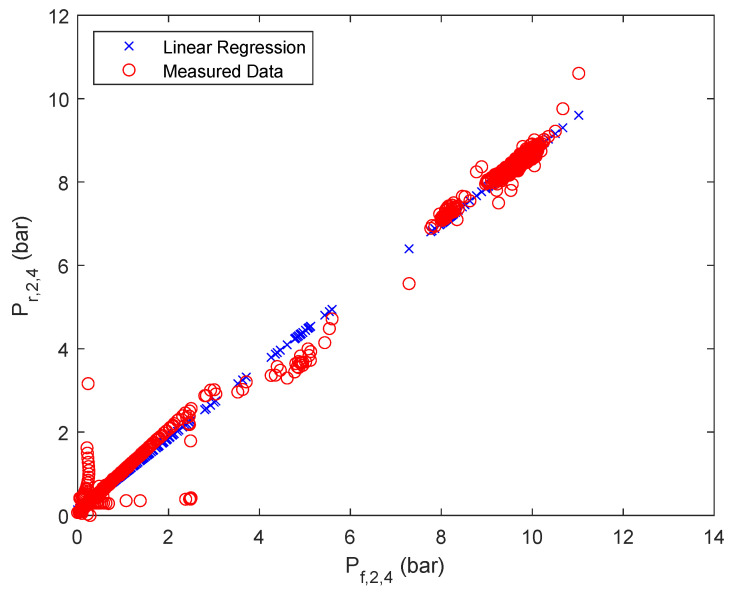
Linear regression results of retentate pressure of stage 2, fourth parallel flow.

**Figure A7:**
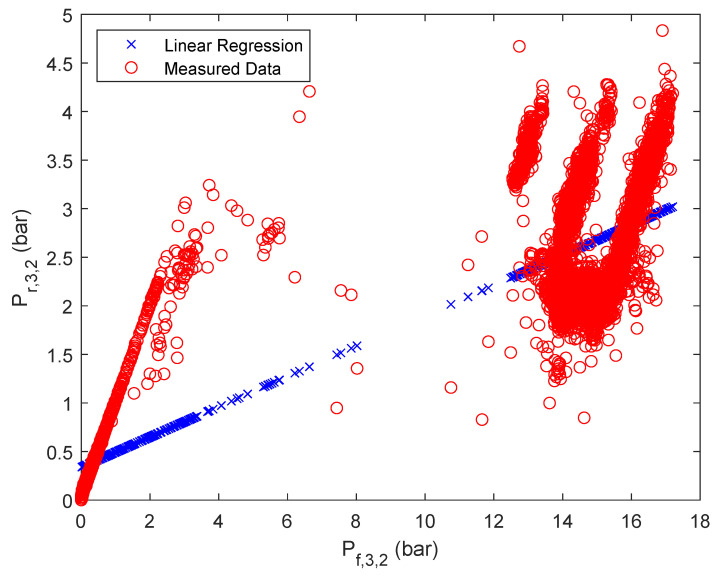
Linear regression results of retentate pressure of stage 3, second parallel flow.

## Data Availability

The data of this study can be provided by the authors upon request.

## Abbreviations

The following abbreviations are used in this manuscript:

ANN	Artificial neural network
BWRO	Brackish water reverse osmosis
ERD	Energy recovery device
FEWN	Food-energy-water nexus
GPM	Gallons per minute
MILP	Mixed-integer linear programming
ML	Machine learning
PSI	Pounds per square inch
RO	Reverse osmosis
SAWS	San Antonio Water System
SEC	Specific energy consumption
TDS	Total dissolved solids

## Symbols

The following Symbols are used in this manuscript:

*Q*	Volume flow	$\frac{m^{3}}{h}$ ($\frac{\mathrm{gal}}{\min}$)
WR	Water recovery	%
*P*	Pressure	bar (psi)
ΔP	Pressure difference	bar (psi)
*C*	Concentration	$\;\frac{\mathrm{mg}}{L}$ ($\frac{\mu S}{\mathrm{cm}}$)
*R*	Root mean square error	-
*W*	Weight of an ANN node	-
*b*	Bias of an ANN node	-
*x*	Positive ReLU output	-
*s*	Negative ReLU output	-
*z*	Binary variable to distinct between positive and negative ReLU outputs	-
*L*	Lower boundary	-
*U*	Upper boundary	-
$\eta_{1}$	Pump efficiency	-
$\eta_{2}$	ERD efficiency	-

## Indices

The following Indices are used in this manuscript:

*f*	Feed
*p*	Permeate
*r*	Retentate
sys	System (water recovery specific)
sum	Overall system value (permeate concentration specific)
primary	Primary RO train
max	Maximum possible value
res	restriction (bound on a variable)
*h*	input factors of hidden layer h∈{1,2,3,4,5}
*i*	number of stages i∈{1,2,3}
*j*	number of parallel flows j∈{1,2,3,4}
*k*	number of ANN layers k∈{1,2}
*l*	number of nodes in hidden layer l∈{1,2,3}
ERD	Energy recovery device

## Appendix A

**Table A1:** Inorganic feed water composition of the RO plant. Comparison of taken measurements in January and July 2017.

Analyte	Results January 2017 ($\frac{\mathrm{mg}}{L}$)	Results July 2017 ($\frac{\mathrm{mg}}{L}$)
Total Alkalinity as CaCO${}_{3}$	222	227
Total Dissolved Solids	1300	1350
Chloride	237	243
Fluoride	0.215	Not measured
Nitrate	<0.5	Not measured
Phosphate	<0.1	Not measured
Sulfate	462	481
Calcium	26.8	24.4
Iron	0.216	0.171
Magnesium	12.5	11.0
Silicon	7.60	8.15
Sodium	418	416
Strontium	2.17	1.96
Iron Dissolved	<0.125	0.159
Aluminum	$13.9\times 10^{-3}$	$14.1\times 10^{-3}$
Barium	$32.8\times 10^{-3}$	$31.5\times 10^{-3}$
Hardness (Ca/Mg calculation)	118	106
Silica as SIO${}_{2}$, Total	16.3	17.4

**Table A2:** Normalized liner regression results for the retentate pressure of Stage 1.

Stage 1				
Parallel Flow	1	2	3	4
Slope	0.9738	0.9961	0.9639	0.9394
Intercept	−0.0178	−0.0109	−0.0159	−0.0429

**Table A3:** Normalized liner regression results for the retentate pressure of Stage 2.

Stage 2				
Parallel Flow	1	2	3	4
Slope	0.9428	0.9717	0.9387	0.8924
Intercept	−0.0438	0.0002	−0.0344	−0.0821

**Table A4:** Normalized liner regression results for the retentate pressure of Stage 3.

Stage 3		
Parallel Flow	1	2
Slope	0.5704	0.5551
Intercept	−0.2841	−0.3053

**Table A5:** Linear regression performance for the retentate pressure of each stage and parallel flow, in terms of R (root mean square error).

Parallel Flow	Stage 1	Stage 2	Stage 3
1	0.9996	0.9997	0.9744
2	0.9965	0.9997	0.9746
3	0.9997	0.9999	X
4	0.9996	0.9998	X

**Table A6:** Weights and biases of the ANN to approximate $\Delta P_{out}^{ERD}$. One input ($P_{f,3,j}$) and one hidden layer with three nodes.

Layer	Weight	Bias
Hidden layer	0.7617	−0.2093
	−0.9346	0.6925
	0.7775	0.3956
Output layer	−0.5216	
	−0.4094	−0.3413
	1.2498	

**Table A7:** Weights and biases of the ANN to approximate $P_{out,j}^{ERD},\;\forall j=\left\{1,2\right\}$. One input ($P_{f,3,j}$) and one hidden layer with two nodes.

Layer	Weight	Bias
Hidden layer	−1.7695	−1.3021
	1.7932	−1.3353
Output layer	−2.0706	−0.017
	2.1311	

**Table A8:** Weights and biases of the ANN to approximate $C_{p,sum}$. Five inputs ($Q_{f}$, $WR_{1}$, $WR_{2}$, $WR_{3}$, $C_{p,t-1}$) and one hidden layer with three nodes.

Layer	Weight	Bias
Hidden layer	−0.9282 0.1619 1.1833 −0.7842 1.2263	0.2516
	−0.0198 −0.0470 0.0774 0.0187 −1.4914	−0.4574
	−0.6438 1.0768 −2.2842 0.9856 0.7062	−0.5085
Output layer	−0.2997	
	−0.5658	−0.3704
	1.8043	
